# EBNEO Commentary: Does High Oxygen Intact Cord Ventilation Improve Early Oxygenation in Preterm Infants?

**DOI:** 10.1111/apa.70447

**Published:** 2026-01-14

**Authors:** Simone Pratesi

**Affiliations:** ^1^ University of Florence, Careggi University Hospital Florence Italy

## Abstract

100% oxygen with the cord intact improves early oxygenation in preterm infants.

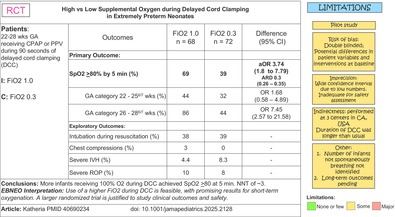

## 1 Manuscript Citation

Katheria AC, Ines F, Lee HC, et al. Deferred Cord Clamping With High Oxygen in Extremely Preterm Infants: A Randomised Clinical Trial. *JAMA Pediatr*. 2025 Sep 1;179 (9):971–978. doi:10.1001/jamapediatrics.2025.2128.

## 2 Commentary

This is a very interesting and well‐designed trial with several strengths. Deferred cord clamping (DCC) for at least 120 s is associated with the highest likelihood of reducing mortality in preterm newborns [1]. However, such prolonged DCC is feasible only when the newborn does not require respiratory support within the first minute of life.

Recently, three randomised controlled trials have compared resuscitation with an intact cord (lasting 2 to 6 min) to either deferred cord clamping (30–60 s) or umbilical cord milking [2, 3, 4]. All three trials failed to show an improvement in the primary outcome. This raises an important question: why does intact cord resuscitation not improve outcomes in preterm infants compared to resuscitation after DCC or milking?

Possible explanations include reduced resuscitation quality at the bedside (with greater hypothermia), technical difficulty and variable expertise [5], and persistent early hypoxia, with low oxygen saturation at 5 min of life linked to adverse outcomes in very preterm infants [6].

Recent animal studies provide new physiological insights [7, 8]. In preterm lambs, brief ventilation with 100% oxygen while the umbilical cord remains intact promotes an increase in pulmonary blood flow without inducing systemic hyperoxia [7]. This probably occurs because the newborn reduces oxygen uptake across the placenta, and when oxygen levels exceed foetal needs, the mother effectively acts as an ‘oxygen sink’ [8].

In the current trial, a peripheral oxygen saturation of 80% at 5 min was achieved more frequently (69% vs. 39%) when preterm infants were resuscitated on the cord with 100% oxygen for up to 90 s, compared to 30 s. This may reflect the beneficial role of 100% oxygen in enhancing pulmonary vasodilation and blood flow, as seen in lambs, and potentially in facilitating glottic opening and pulmonary ventilation. However, the similar rates of positive pressure ventilation (PPV) before and after cord clamping, along with comparable intubation rates between the low‐ and high‐oxygen groups, indicate that the beneficial effects of oxygenation on the initiation of spontaneous breathing or glottic opening do not appear to translate into clinical advantages. The two following considerations could at least partially explain this result. The paper fails to indicate how many infants were not breathing after birth, and this subgroup might have benefited more from receiving 100% oxygen. This is a critical consideration, especially since the Vent First trial [2] reported a high proportion (48%) of infants who were not breathing adequately at birth, and this subgroup appeared to benefit more from intact cord resuscitation with 30% oxygen. Moreover, it should be kept in mind that clinicians often underestimate spontaneous breathing and are frequently quick to initiate PPV in preterm infants, which makes it more difficult to detect differences in PPV rates between groups [9].

Administering 100% oxygen with the cord intact improves early oxygenation in preterm infants without increasing the risk of short‐term morbidity. Future larger trials on intact cord resuscitation, conducted in centres with established expertise in this procedure, should incorporate the short‐term use of 100% oxygen into their study design. This may help demonstrate improved outcomes in infants resuscitated with an intact cord.

URL LINK: https://ebneo.org/ebneo‐commentary‐high‐o2‐intact‐cord‐ventilation‐and‐early‐oxygenation.

## Funding

The author has nothing to report.

## Conflicts of Interest

The author declares no conflicts of interest.

## Data Availability

The data that support the findings of this study are available from the corresponding author upon reasonable request.
